# Genome Editing Redefines Precision Medicine in the Cardiovascular Field

**DOI:** 10.1155/2018/4136473

**Published:** 2018-03-14

**Authors:** Elda Dzilic, Harald Lahm, Martina Dreßen, Marcus-André Deutsch, Rüdiger Lange, Sean M. Wu, Markus Krane, Stefanie A. Doppler

**Affiliations:** ^1^Department of Cardiovascular Surgery, German Heart Center Munich, Technische Universität München, Lazarettstraße 36, 80636 Munich, Germany; ^2^Insure (Institute for Translational Cardiac Surgery), Department of Cardiovascular Surgery, German Heart Center, Technische Universität München, Lothstraße 11, 80636 Munich, Germany; ^3^German Center for Cardiovascular Research (DZHK), Partner Site Munich Heart Alliance, Munich, Germany; ^4^Department of Medicine, Division of Cardiovascular Medicine, Cardiovascular Institute, and Institute for Stem Cell Biology and Regenerative Medicine, Stanford University, 265 Campus Drive, Stanford, CA 94305, USA

## Abstract

Genome editing is a powerful tool to study the function of specific genes and proteins important for development or disease. Recent technologies, especially CRISPR/Cas9 which is characterized by convenient handling and high precision, revolutionized the field of genome editing. Such tools have enormous potential for basic science as well as for regenerative medicine. Nevertheless, there are still several hurdles that have to be overcome, but patient-tailored therapies, termed precision medicine, seem to be within reach. In this review, we focus on the achievements and limitations of genome editing in the cardiovascular field. We explore different areas of cardiac research and highlight the most important developments: (1) the potential of genome editing in human pluripotent stem cells in basic research for disease modelling, drug screening, or reprogramming approaches and (2) the potential and remaining challenges of genome editing for regenerative therapies. Finally, we discuss social and ethical implications of these new technologies.

## 1. Introduction

The human genome project was a breakthrough for the scientific world. Knowing the sequence of the human genome allows the study of the function of specific genes or proteins [1]. Unfortunately, the initial methods at hand were inefficient to robustly identify the role of certain genes and proteins. However, genome editing with engineered nucleases offers the opportunity to insert or delete DNA sequences in a very reliable and elegant manner. Hence, by creating knock-out or knock-in models, gene and protein function can be easily and reliably investigated. The most promising genome editing technologies are zinc finger nucleases (ZFNs), transcription activator-like effector nucleases (TALEN), and clustered regularly interspaced short palindromic repeats- (CRISPR-) associated protein 9 (Cas9). Especially the recently developed CRSIPR/Cas9 technology revolutionized the field of genome editing. In 2015, CRSIPR/Cas9 was even selected by science as breakthrough of the year [2]. Many excellent reviews are available describing the above-listed genome editing tools in detail [3–5].

In general, the above-mentioned genome editing tools can be designed to induce double-strand breaks (DSB) at almost any specific genomic location desired, leading to the activation of the cellular endogenous repair machinery—nonhomologous end-joining (NHEJ) and homologous directed repair (HDR). NHEJ is an imprecise repair mechanism that can lead to random insertions and deletions (indel mutations) at the site of the DSB. This can result in frameshift mutations or premature stop codons, leading to a potentially dysfunctional protein. By contrast, by recombination with the second allele, HDR allows the exact repair of the DSB. However, by introducing a donor sequence functioning as a repair template, tailored modifications can be inserted into the endogenous sequence. A limiting factor, especially for the cardiovascular field since cardiomyocytes are largely postmitotic cells, is the fact that HDR is solely occurring in dividing cells, but first attempts are being developed to overcome this obstacle [6, 7]. This genome editing mechanism, the accuracy, and ease-of-use of the new technologies have enormous potential for basic science as well as for regenerative medicine. Nevertheless, there are still several hurdles that have to be overcome, but precision medicine seems to be within reach.

In this review, we focus on the achievements and limitations of genome editing in the cardiovascular field. We explore different areas of cardiac research and highlight the most important developments: (1) the potential of genome editing in human pluripotent stem cells in basic research for disease modelling, drug screening, or reprogramming approaches and (2) the potential and challenges of genome editing for regenerative therapies. Finally, we discuss the social and ethical implications of these new technologies.

## 2. Genome Editing in Basic Research: Human Pluripotent Stem Cells and Beyond

The pioneering method to reprogram murine and especially human fibroblasts into induced pluripotent stem cells (iPSCs) introduced by Takahashi et al. revolutionized stem cell research [8, 9]. Thus, not only the ethical problems of human embryonic stem cells (hESCs) have been overcome, but the possibility to produce patient-specific cells is a breakthrough in the field of basic research. However, genome editing allows researchers to tap the full potential of human iPSCs (hiPSCs).

### 2.1. Disease Modelling

**Figure 1:**
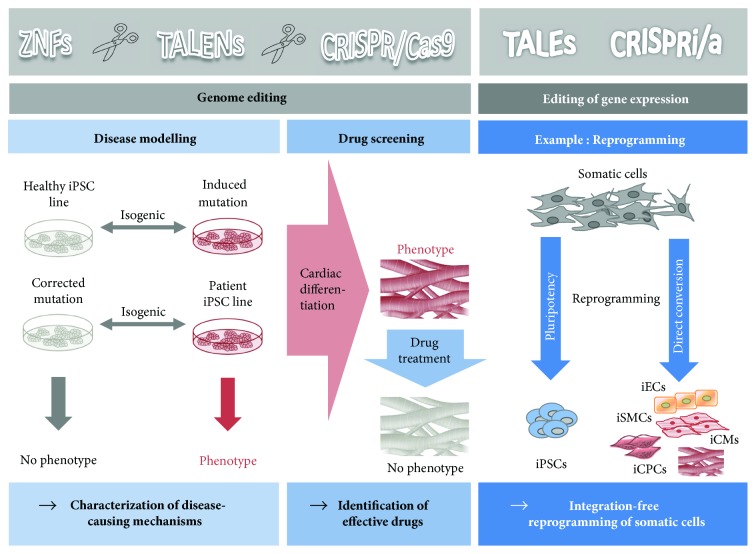
Genome editing approaches in basic research. In basic research, genome editing tools find broad utilization. ZNFs, TALENs, and CRISPR/Cas9 allow genome editing in human pluripotent stem cells in basic research for disease modelling, drug screening, or even the editing of gene expression, for example, for reprogramming approaches. This might help in the characterization of disease-causing mechanisms, the identification of new effective drugs, or the development of innovative regenerative approaches by an integration-free reprogramming/transdifferentiation of somatic cells into another cell type. ZNFs: zinc finger nucleases; TALENs: transcription activator-like effector nucleases; CRISPR/Cas9: clustered regularly interspaced short palindromic repeats; TALEs: transcription activator-like effector protein; CRISPRi: CRISPR interference; CRISPRa: CRISPR activation; iPSCs: induced pluripotent stem cells; iECs: induced endothelial cells; iSMCs: induced smooth muscle cells; iCMs: induced cardiomyocytes; iCPCs: induced cardiac progenitor cells.

Ever since the possibility to create patient-specific hiPSCs the research on *in vitro* disease modelling boomed. The iPSC technology allows investigating complex pathophysiological mechanisms directly in human cellular models. However, over the years, the validity of the collected data was repeatedly questioned. As it is well known, a study is only as good as its control group. The standard control groups in the first disease modelling studies were hiPSCs from healthy relatives or random individuals without the disease. Hence, the observed phenotypical differences may potentially be due to the different genetic backgrounds and other confounders, rather than disease-specific mutations. Therefore, the ideal comparison would be between two lines that only differ in the supposed disease-specific mutation and are otherwise genetically matched (isogenic lines). Genome editing technologies allow the production of isogenic lines by offering the possibility to preciously introduce or correct a mutation. Thus, the observed phenotypical differences can be unequivocally assigned to the genotype (Figure 1).

One of the first studies, using genome editing in hiPSCs to study cardiovascular diseases investigated the Barth syndrome, a mitochondrial disorder caused by mutation of the gene tafazzin (TAZ) [10]. Wang et al. introduced a patient-specific TAZ mutation into a healthy iPSC line using CRISPR/Cas9. After differentiation into cardiomyocytes (CMs), the group was able to confirm the previously established phenotype from the patient-specific iPSC-derived CMs in the newly developed, diseased line. The isogenic healthy control line did not display the same phenotype, confirming that the abnormalities were caused by the mutation. Unfortunately, the group did not correct the mutation in the patient-specific iPSC line by genome editing. They used TAZ mRNA to restore TAZ function and were at least able to demonstrate a partially rescued phenotype.

Around the same time, Karakikes et al. described the generation of hiPSCs with a mutation in the coding region of the phospholamban gene (R14del) that is associated with cardiomyopathy, ventricular dilation, ventricular arrhythmias, and heart failure [11]. The investigators used TALENs to correct the R14del mutation and were able to show that the abnormalities in calcium handling and the abnormal cytoplasmic distribution of the phospholamban could be rescued after gene correction.

Since then, other groups studying cardiovascular diseases used genome editing to create isogenic lines in order to eliminate any potential confounders [12–15]. So far, all of the mentioned publications either introduced a known mutation in an unaffected iPSC line or corrected a mutation in a patient-specific iPSC line. Ideally, these two approaches need to be combined in order to create two pairs of isogenic lines that allow a distinct, undoubted confirmation of the supposed disease-causing mutation: (1) patient-specific iPSCs, (2) isogenic patient-specific iPSCs with the corrected mutation, (3) healthy control iPSCs, and (4) isogenic control iPSC with the inserted mutation (Figure 1). One impressive example for creating two pairs of isogenic iPSC lines is the study from Bellin et al. [16]. The investigators studied the role of a potassium voltage-gated channel subfamily H member 2 mutation in long QT syndrome by generating such isogenic iPSC lines. They were able to robustly trace back the mutation to the phenotypical abnormalities. The correction and insertion of the mutation were not performed by the new genome editing tools though, but by using bacterial artificial chromosome vectors. Nevertheless, this study can serve as a model on how to design valid study groups. Gupta et al. took another approach in their study about reverse cholesterol transport in macrophages [17]. The investigators used NHEJ to knock-out ATP-binding cassette, subfamily A, member 1 (ABCA1), which is a major player in the process of reverse cholesterol transport. By introducing random mutations resulting in a loss of function of ABCA1, the group was able to show reduced reverse cholesterol transport. This study demonstrates that genome editing enables researchers to investigate diseases without the need of recruiting patients. In terms of precision medicine, this allows us to study rare disease where patient recruitment might be harder due to logistical problems. However, genome editing offers the opportunity to easily introduce a known disease-specific mutation in order to study the pathophysiology. Importantly, it needs to be stressed that patient-specific iPSCs are to be preferred compared to disease-specific iPSCs, because the clinical phenotype of the genetic disorder is confirmed.

The simplicity, speed, and cost effectiveness of the new genome editing technologies allow the study of cardiovascular diseases on a large scale. Recently, Karakikes et al. created a TALEN-based knock-out library targeting 88 different genes associated with cardiovascular diseases, including CHARGE syndrome, Leigh syndrome, Holt-Oram syndrome, Noonan syndrome, and LEOPARD syndrome [18]. Notably, the investigators created a cell line modelling Holt-Oram syndrome, a congenital disorder characterized by structural cardiac and limb abnormalities, by introducing a mutation into the T-box protein 5 (TBX-5) gene. By using a TALEN pair targeting the start codon at exon 1 of the major isoforms of the gene, they were able to identify a clone that resulted in an early termination of the gene leading to electrophysiological changes like proarrhythmic activity of the diseased hiPSC-CMs. This TALEN library represents a great resource for the further study of cardiovascular diseases. Readily available constructs like these allow the effortless development of iPSCs mimicking monogenetic disorders. Additionally, by using more than one construct in one iPSC line, even complex diseases can be modelled.

### 2.2. Drug Screening

Patient-specific hiPSCs not only allow investigating the pathophysiology of the underlying disorder but also provide a platform for drug screening and drug development (Figure 1). So far, the lack of certain animal disease models hindered the progress in this field of research, but genome editing provides a limitless source for generating new disease-specific iPSCs.

As proof of concept, Wang et al. created a transgenic iPSC line using the ZFN technology recapitulating the long QT syndrome (LQTS) phenotype [19]. After differentiating the genome-edited diseased hiPSCs into cardiomyocytes, the conducted electrophysiological analysis showed a prolongation of the action-potential duration as expected for the LQTS disorder. The cardiomyocytes derived from the isogenic healthy control line displayed a normal action-potential duration. The investigators established the genome-edited line as a platform for drug screening by treating iPSC-derived cardiomyocytes with nifedipine and pinacidil. Both drugs are known to shorten the action-potential duration, which was also confirmed in the patch clamp assay performed on the disease-specific iPSC-derived cardiomyocytes.

LQTS and also other cardiovascular diseases are caused by different mutations either in the same gene or in different genes. Clinical experience taught us that such variations may alter the patient's response to certain treatments. Genome editing gives the opportunity to create disease-specific as well as patient-specific iPSCs with different known mutations and use them as a platform for drug screening and even drug discovery or development. Hence, the treatment can be tailored to the genetic background of each individual patient. Prescribing the most effective drug for each patient offers the chance to minimize side effects, to increase patients' compliance, and to reduce health care costs.

However, hiPSC-derived cardiomyocytes still have their limitations as a drug discovery platform. Most importantly, hiPSC-derived cardiomyocytes are structurally and functionally immature compared to cardiomyocytes from adult human hearts [20]. They are small, round, mononuclear cells with a disorganized sarcomeric structure and no T-tubules. Their electrophysiological features show lower maximum diastolic potential and slower maximum rate of depolarization. The gene expression pattern of hiPSC-derived cardiomyocytes resembles human fetal cardiomyocytes. But extensive research is being conducted on strategies to assist physiological maturation of hiPSC-derived cardiomyocytes. Physical, chemical, electrical, and genetic factors are being tested as stimuli for further maturation [21].

Another major limitation in conducting accurate drug screening and drug development studies lies in the fact that directed differentiation into cardiomyocytes produces a mixture of ventricular, atrial, and nodal subtypes. These cardiomyocyte subtypes display different structural and functional properties and therefore respond differently to the same drug. In order to get clear and robust data, drug screening needs to be performed on the isolated subtype of interest. First attempts are being made to purify for a specific cardiomyocyte subtype, using beacon-based detection [22], based on different subtype-specific surface marker expression [23], or by creating subtype-specific reporter lines [24–26].

### 2.3. Editing Gene Expression by Genome Editing Tools

TALENs and CRISPR/Cas9 are more than mere genome editing tools. By inactivating the nuclease function of these technologies, they are still able to bind DNA but are not able to cleave it. Utilizing these new tools allows altering the gene expression by basically acting like transcription factors [27]. Designing the complexes to bind at promoter regions blocks the transcription initiation and consequently the gene expression with up to 1000-fold repression leading to 90%–99% gene knockdown (CRISPR interference, CRISPRi; transcription activator-like effector protein, TALE) [28]. The major benefit of this technology is the reduced off-target binding compared to the previously used RNA interference (RNAi) method, which is described to have up to 10% off-target effects [29, 30]. Additionally, CRISPR activation (CRISPRa) and TALEs can also be utilized to initiate gene expression by fusing a transcriptional activator to the deactivated nuclease, which binds to the promoter region of the target gene and activates transcription. Common methods used hitherto to activate gene expression involved in the transfection of viral vectors, which could be avoided with the new-adapted genome editing tools. Different groups used these engineered transcription factors to modulate the gene expression in order to activate pluripotency genes in somatic cells, ultimately trying to “reprogram” them into iPSCs [31–37] (Figure 1). So far, successful reprogramming using CRISPRi/a or TALEs has not been achieved, but the first results sound promising. The initial methods for reprogramming using retro- or lentiviral vectors were soon outdated by nonintegrating techniques. Especially the nonintegrative Sendai virus has been widely used, but residual viral material persists up to 10–12 passages [38]. Future clinical applications of hiPSCs require safe, nonintegrative, vector-free reprogramming techniques. However, CRISPRi/a and TALEs need to be further investigated in order to potentially qualify as safer alternative.

Besides reprogramming back to pluripotency, Chakraborty et al. were able to use CRISPRa for direct reprogramming/transdifferentiation of somatic cells without passing through a pluripotent state [39] (Figure 1). The investigators were able to directly reprogram mouse embryonic fibroblasts into skeletal myocytes by activating the endogenous Myod1 gene locus with CRISPRa. However, the conversion of a human embryonic kidney fibroblast cell line (HEK293T) to skeletal myocytes seems not to be possible due to low activation of human Myod1. The investigators showed that the activation of human Myod1 in HEK293T cells was an order of magnitude lower compared to the transgenic expression level induced in mouse embryonic fibroblasts. They hypothesized that this expression level is not sufficient for successful transdifferentiation of human fibroblasts. Though not successfully implemented yet, these studies show the potential of the new genome editing tools for reprogramming somatic cells back to pluripotency or for direct reprogramming approaches. A direct conversion of cardiac resident fibroblasts into cardiomyocytes or other cardiac lineages *in vivo* after injury is an important field of research and one of the most promising regenerative strategies, and CRISPRi/a and TALEs may have a potential value to move the field into the right direction.

## 3. Regenerative Medicine in the Era of Genome Editing Is within Reach

**Figure 2:**
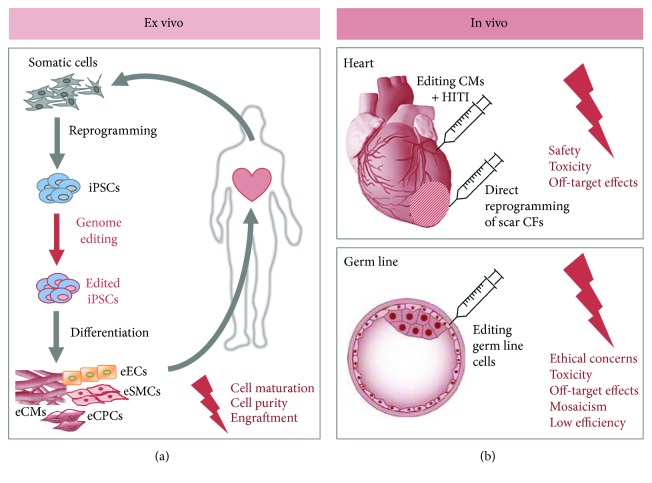
Genome editing for regenerative medicine. The future application of genome editing techniques *in vivo* for regenerative therapies in the cardiovascular field is still in the early stages of development. This figure shows the potential and remaining challenges of genome editing for regenerative therapies. One option is to produce iPSCs from a patient and edit the gene of interest ex vivo (a). After editing the iPSCs, they can be expanded, differentiated into the desired cell type, and transplanted back into the patient. Remaining problems are mainly cell maturation and purification issues as well as low engraftment after transplantation. The other option is *in vivo* genome editing by directly targeting the gene of interest in the host organism. With the implementation of homology-independent targeted integration (HITI), precise genome editing is even possible in nondividing cells like cardiomyocytes (b). However, besides safety and toxicity issues, off-target effects have to be entirely excluded before clinical application. Many genetic diseases cannot be cured with targeting somatic cells, thereby demanding the use of germ-line editing. But genome editing in human embryos is of course highly controversial, so that safety and ethical concerns need to be fully addressed before moving on to clinical application. iPSCs: induced pluripotent stem cells; eECs: edited endothelial cells; eSMCs: edited smooth muscle cells; eCMs: edited cardiomyocytes; eCPCs: edited cardiac progenitor cells; CMs: cardiomyocytes; CFs: cardiac fibroblasts; HITI: homology-independent targeted integration.

Mason and Dunnill defined regenerative medicine in 2008 as follows: “Regenerative medicine replaces or regenerates human cells, tissue or organs, to restore or establish normal function” [40]. Obviously, stem cells had a great impact on regenerative medicine leading to the development of new exciting treatments [41, 42]. With the advancements in genome editing, promising trails are blazed on the path to curing genetic disorders. However, one has to be aware that significant barriers remain for a translation of genome editing technologies to a therapeutic clinical application, particularly concerning safety and toxicity issues. Nevertheless, genome editing opens up new dimensions of possibilities, especially in terms of gene therapy, which can be achieved either *in vivo* by direct delivery of the nucleases via injection or ex vivo by editing the cells in the dish before autologous transplantation (Figure 2).

### 3.1. Ex Vivo Approaches

As mentioned earlier, HDR only operates in dividing cells; consequently, this repair mechanism is not present in the mainly postmitotic mature cardiomyocytes. One way to circumvent this problem is to produce iPSCs from the patient and edit the gene of interest ex vivo. After confirmation of the correct editing, the edited iPSCs can be expanded, differentiated into the desired cell type, and transplanted back into the patient. Besides cardiomyocytes, endothelial cells, smooth muscle cells, or cardiac progenitor cells, which are able to develop into all cardiac lineages, would be desired cell types for cardiac regeneration (Figure 2). This new technique is still in its infancy and has not, as yet, been implemented into the cardiovascular field as a whole. However, experiences from stem cell work and a glimpse into other fields are going to allow rapid progress.

Previous work illustrated that it is crucial to transplant a pure population of differentiated cells and make sure no residual iPSCs are left in order to prevent tumorigenesis. Extensive research and the implementation of purification methods have already allowed the safe transplantation of unedited stem cell-derived cardiomyocytes [43–45]. However, cell purity, especially in the cardiovascular field, is more than just the lack of residual iPSCs. As mentioned earlier, differentiation into cardiomyocytes produces a cell mixture of different subtypes, ventricular, atrial, and nodal CMs including cardiomyocytes with different maturation states. The transplantation and potential engraftment of nodal cardiomyocytes into the myocardium can lead to severe arrhythmias. Recently, Shiba et al. published their work on transplantation of iPSC-derived-cardiomyocytes after myocardial infarction in cynomolgus monkeys whose major histocompatibility complex structure is identical to that of humans [46]. The investigators observed a higher rate of arrhythmias after transplantation, stressing the need for maturation and perhaps subtype-specific purification of cardiomyocytes in order to eliminate the transplantation of arrhythmogenic cells.

Another concern of the ex vivo approach is the debatable efficiency of cell engraftment into the host myocardium. Freyman et al. evaluated the engraftment of mesenchymal stem cells after intravenous, intracoronary, or endocardial delivery in a porcine model of myocardial infarction [47]. The comparison showed that the engraftment of the transplanted cells was highest after intracoronary injection, followed by endocardial delivery. After intravenous injection, there was no engraftment detectable at all. It has to be noted that even the intracoronary injection only achieved 6% of cell engraftment of the total administered dose which is far from sufficient.

One way to avoid issues of low cell engraftment is by making use of the new developments in the field of tissue engineering, namely, patch-based approaches. Recently, Menasché et al. performed the first clinical transplantation of human embryonic stem cell-derived cardiac progenitor cells embedded into a fibrin scaffold [48]. The SSEA-1-positive cardiac progenitor cells strongly express the early transcription factor Islet1 and should be able to develop in different cardiac lineages like cardiomyocytes, endothelial cells, and smooth muscle cells. The investigators implanted the tissue-engineered construct into a patient with heart failure undergoing coronary bypass surgery. They created a pocket between the pericardial flap and the epicardium across the infarction area for the cell-loaded patch. After six months, they found no tumor growth and no occurrence of arrhythmia. The patient's left ventricular function improved substantially, but this effect is maybe due mostly, if not entirely, to the beneficial effect of the bypass surgery. Nevertheless, this trial is a major landmark on the way to implementing regenerative medicine into clinical practice.

The studies outlined here did not use genome editing in order to treat cardiovascular diseases, but they provide useful data on the way to apply ex vivo approaches of genome editing to cardiovascular patients.

### 3.2. *In Vivo* Approaches

Precise *in vivo* genome editing in cardiovascular disorders is challenging due to the nonoccurrence of HDR in nondiving cells like mature cardiomyocytes. Recently, Suzuki et al. published a method enabling a specific modification of the endogenous sequence, even in nondividing cells [49]. The investigators developed a new genome editing tool based on CRISPR/Cas9, which makes use of the NHEJ repair mechanism: homology-independent targeted integration (HITI). The HITI donor construct is a circular vector that can be integrated either in the forward or in the reverse direction at the site of the DSB whereby the forward integration was proven to occur more frequently. Reverse or no integration of the vector allows repeated Cas9 cutting as long as the sgRNA sequence stays intact. Suzuki and his coworkers were able to show that the genome editing efficiency via HITI is approximately ten times higher than HDR. This innovative method allows precise, tailored *in vivo* genome editing in cardiomyocytes, opening up new opportunities for treatment of genetic cardiovascular diseases (Figure 2).

Some cardiovascular diseases do not require precise genome modifications though. Experiences using 2′-O-methyl phosphorothioate- (2OMePS-) antisense oligoribonucleotides (AONs) showed that imprecise exon skipping can restore gene function [50, 51]. Gedicke-Hornung et al. were able to recover the function of the mutated MYBPC3 gene encoding cardiac myosin-binding protein C, which is frequently mutated in hypertrophic cardiomyopathy, by exon skipping via RNA modulation using AONs [51]. However, RNA modulation is only transient, so genome editing paves the way for a more definitive way of treatment.

As a proof of concept, NHEJ-mediated exon skipping was performed in Duchenne muscular dystrophy. Duchenne is the most common severe form of muscular dystrophy in childhood, which also affects cardiac muscle resulting in heart failure by the age of twenty. Three separate groups published their work on exon skipping of the mutant dystrophin exon in neonatal or adult mdx (X chromosome-linked muscular dystrophy) mice which have a point mutation in the dystrophin gene on the X chromosome resulting in a truncated, dysfunctional protein and leading to a mild form of Duchenne [52–54]. The investigators utilized the CRISPR/Cas9 system to skip exon 23 and thereby partially restored the dystrophin protein function. The Cas9 and sgRNA vectors were delivered by adeno-associated virus by intramuscular, retroorbital, and intraperitoneal injection. All three groups were able to confirm functional and histological recovery of the dystrophin-positive fibers, including cardiomyocytes. These studies provide encouraging data that the clinical application of genome editing in cardiovascular disorders is feasible.

Besides restoring gene function, NHEJ can also be used to disrupt gene function and thereby treat cardiovascular disorders. Ding et al. were able to prove this concept in their study about proprotein convertase subtilisin/kexin type 9 (PCSK9) [55]. PCSK9 plays a role in low-density lipoprotein- (LDL-) cholesterol clearance by functioning as an antagonist to the LDL-receptor. A mutation resulting in loss of function of PCSK9 leads to significant reduction in LDL-cholesterol levels [56]. The investigators used adenoviruses to deliver CRISPR/Cas9 into the liver of adult mice leading to a mutagenesis rate of PCSK9 of up to 50%. Laboratory testing revealed a 35 to 40% decrease of blood cholesterol.

Another way to apply *in vivo* genome editing to treat cardiovascular disorders is to utilize resident cardiac fibroblast as a source to generate cardiomyocytes or other cardiac lineages by direct reprogramming. Fibroblasts account for up to 50% of cardiac cells, and more importantly, they expand after myocardial infarction in the infarct zone generating scar tissue [57]. This approach would allow the generation of functional myocardium after injury leading to an improvement of cardiac function. As described above, direct reprogramming using genome editing technologies has not been implemented as yet, but the progress in this field of research is promising.

Many genetic cardiovascular disorders go along with structural and functional changes during embryogenesis. Targeting the mutation after birth would hardly improve the patient's condition, but genome editing in human embryos is highly controversial. Nevertheless, Liang et al. edited human embryos with CRISPR/Cas9 targeting a mutation in the *β*-globin gene [58]. The mutation is known to cause *β*-thalassemia, an inherited blood disorder that can lead to anemia. The investigators used human tripronuclear zygotes generated for *in vitro* fertilization, which have one oocyte nucleus and two sperm nuclei and are not suitable for clinical transfer. After injection of the mRNA and DNA needed for CRISPR and HDR, 80% of the embryos survived, indicating low, but still present toxicity after injection. The survived embryos showed 52% on-target efficiency with a 14.3% HDR rate, and additionally, some of the embryos displayed mosaicism. Six randomly selected embryos were selected for whole-exon sequencing, whereby two of the embryos showed one off-target effect in sequences similar to the sgRNA sequence. Recently, Tang et al. published their work on genome editing dual pronuclear zygotes using CRISPR/Cas9 [59]. First, the investigator edited zygotes carrying a *β*-thalassemia causing mutation in the *β*-globin gene. The investigators observed a 50% on-target efficiency with a 50% HDR rate. Furthermore, they edited two zygotes with a glucose-6-phosphate dehydrogenase deficiency, known as favism, which may lead to hemolysis. The HDR rate turned out to be 100%, whereas one of the embryos was mosaic. Whole-genome sequencing of the nonmosaic embryo revealed seven potential off-target sites. However, these sites are known SNPs. Although it seems that CRISPR/Cas9 has higher HDR rates in normal, dual pronucleus compared to tripronuclear zygotes, off-target effects and mosaicism were still observed in both studies.

A major breakthrough in regard to embryonic genome editing was recently published by Ma et al. [60]. The group was able to successfully edit human zygotes with a heterozygous mutation in the MYBPC3 gene causing hypertrophic cardiomyopathy. The zygotes were created by intracytoplasmic sperm injection (ICSI) of heterozygous, carrier sperm into healthy oocytes carrying the wild-type allele. The CRISPR-Cas9 complex was either injected 18 hours after ICSI or coinjected with the sperm during ICSI. The repair template used for HDR contained two additional single-nucleotide substitutions in order to distinguish the sequence from the wild-type allele. Interestingly, HDR was solely performed using the wild-type allele and not the exogenous DNA as template. The investigators assume that different DNA repair mechanisms are being at work in human embryos compared to somatic or pluripotent cells, leading to preferential use of the second allele as repair template. Also, in contrast to the two above-mentioned studies, Ma et al. did not find any off-target effects caused by introducing CRISPR-Cas9. Furthermore, the study showed that mosaicism can be diminished by coinjecting the sperm and the CRISPR complex at the earliest stage possible. This finding is an important step in bringing genome editing closer to clinical use, but needless to say that ethical consideration concerning embryonic research cannot be ignored.

## 4. Main Challenge of Genome Editing: Off-Target Effects

Besides safety and toxicity issues for *in vivo* approaches, the biggest concern regarding the new genome editing technologies is additional off-target cleavage sites created by imprecise cutting of the nucleases. As described above, one of the main advantages using ZFNs, TALENs, and CRISPR/Cas9 is the possibility to create isogenic cell lines for basic research and by this, it generates the perfect control group for downstream analysis. Having off-target effects lessens this benefit by creating new confounders. One possibility to minimize the effect of potential off-target sites in basic research is to compare the phenotype of the wild-type clone with multiple genome-edited clones. If the phenotypical difference is still observed in all of the clones, the causal link between the mutation and the phenotype can be established.

However, for clinical use of genome-edited cells or tissues, the occurrence of off-target effects must be completely excluded. Unfortunately, there is no reliable way to assess or truly predict the rate of off-target sites. Especially CRISPR/Cas9 seems to be prone to off-target cleavage due to incorrect binding of the sgRNA. Similar DNA sequences trigger sgRNA binding, leading to the activation of Cas9 and DSBs. Tsai et al. were able to show that up to 6 mismatches in the 20 nucleotides (nt) sgRNA sequence and also noncanonical protospacer adjacent motifs (PAMs) are tolerated and can cause off-target cleavage [61]. Several other studies showed high off-target effects of CRISPR/Cas9 ranging from 7 to 58% when evaluating the predicted off-target locations [62–64]. However, the assessments were mainly performed in cancer cell lines where DNA repair mechanisms are not operating properly in tumor cells. Hence, the exact off-target rate in normal cells remains to be clarified.

In contrast, studies in iPSCs showed very low frequencies of off-target effects. Schwank et al. showed in their study targeting the cystic fibrosis transmembrane conductor receptor by CRISPR/Cas9 in human iPSCs off-target effects of predicted locations between 0 and 4% [65]. However, only checking the predicted sites might be not enough, so Wang et al. conducted whole-genome sequencing after genome editing hiPSCs. The observed off-target mutations ranged from 10 to 15 sites, but only 1 to 3 can be lead back to sgRNA-related Cas9 activity due to similarity of the sequences. The authors hypothesize that the other mutations were acquired during iPSC culturing and passaging. Studies show that the mere maintenance of iPSCs can lead to mutation, including point mutations, but also full chromosome aneuploidy [66]. It should be mentioned that the observed off-target effects were not always at the predicted sites, emphasizing the need for whole-genome sequencing after genome editing. Similar to this study, Smith et al. performed whole-genome sequencing using CRISPR/Cas9 and TALENs in hiPSCs [67]. They identified 217 to 281 single-nucleotide variants and 7 to 12 small indels but questioned how many of these mutations arose during the genome editing process. After checking for similarities to the sgRNA and TALEN pairs and comparing to potential, bioinformatically predicted sites, they concluded that all of the mutations are probably random and cannot be assigned to genome editing.

Similarly, studies in whole organisms show lower off-target frequencies compared to the previous studies in cancer cell lines. Studies in mice and cynomolgus monkeys detected no mutations at the predicted off-target sites using CRISPR/Cas9 when generating single-gene mutants by zygote injection [68, 69]. However, the investigators did not perform whole-genome sequencing in order to fully exclude any off-target mutations. However, Mianné et al. checked for off-target effects with whole-genome sequencing [70]. The investigators corrected a mutation in mice causing age-related progressive hearing loss by utilizing CRISPR/Cas9 and were not able to detect any off-target effects.

Nevertheless, extensive research is being performed to find ways to predict and eventually completely avoid off-target effects. Fu et al. reported that using a shorter sgRNA sequence (17-18nts rather than 20nts) for CRISPR/Cas9 led to a lower off-target cleavage frequency [71]. For proof of concept, three different sites in a cancer cell line were targeted comparing the truncated sgRNA with the full-length sgRNA and examining 13 potential off-target sites. They observed a 5000-fold decrease in mutation frequency using the truncated sgRNA-Cas9 complex. *Prima facie*, reducing the length of the sgRNA may sound illogical, but considering the binding energy, this approach makes sense. By this, the shorter sgRNA is more sensitive to mismatches and thus binds less at off-target locations.

Another attempt to reduce off-target effects was introduced by Kleinstiver et al. [72–74]. The investigators hypothesized that the Cas9-sgRNA binding complex may function with a lower energetic binding level resulting in a sufficient on-target but lower off-target efficiency. In order for the Cas9 protein to create a DSB, it needs to bind the sgRNA and the DNA via several binding sites, including direct hydrogen bonds. The investigators hypothesized that by disrupting some Cas9 binding sites, the protein binds and cleaves the DNA only when a perfect match is present, thereby avoiding mismatches, ergo off-target effects. Consequently, they were able to show that an altered Cas9 nuclease is able to cleave the DNA on-target but renders low or no off-target cleavage.

Besides disrupting the binding sites of Cas9, Ran et al. deactivated the catalytic domain of the nuclease creating a nickase [75]. The Cas9 nuclease has two domains, RuvC and HNH, allowing the enzyme to generate a DSB. By inactivating one of these domains via introduction of a mutation, the DNA is only cleaved at one strand, resulting in a single-strand break or so-called nick. In general, nicks are repaired very precisely by using the other allele as repair template, and not being repaired via NHEJ. The combination of two nickases targeting both alleles at the site of interest leads to a DSB that can be repaired with NHEJ or HDR. Hereby, the likelihood of two off-target nicks generating a DSB is substantially decreased. The same concept of single-strand breaks is also established for TALEN (TALENickase) [76] and for ZFN (ZFNickases) [77].

Komor et al. used these Cas9 nickases in order to establish a new way of genome editing, called base editing [78]. The investigators modified the Cas9 by deactivating the RuvC catalytic domain and fusing it with two other enzymes: (1) Cytidine deaminase enzyme, which catalyzes the conversion of cytosine (C) to uracil (U) and (2) Uracil glycosylase inhibitor, which blocks the reversion of the U back to C. By converting C to U, which basically acts like thymine (T), and nicking the nonedited strand, this new fused enzyme leads to a single base pair transformation (C:G to A:T), without the need of introducing a DSB. Recently, the group also modified the enzyme in order to enable the A:T to C:G transformation [79]. With this technology avoiding DSB, they were able to reduce the off-target effects to less than 1%. Unfortunately, this method allows just a limiting number of target sites, due to the dependency of Cas9 to a PAM sequence (NGG locus) and the potential editing of any cytosine within the range of 5 base pairs. After modifying the enzyme, Kim et al. were able to improve the on-target accuracy by increasing the variation number of PAM sequences and reducing the editing range to two or three base pairs [80]. Therefore, base editing seems to be a good and safe option when dealing with single-nucleotide mutations.

Besides this main hurdle of genome editing, there are also other challenges that need to be mentioned. Although HDR is naturally a repair mechanism occurring to a lesser extent than NHEJ, respecting certain design mechanisms in regard to the donor repair template can increase the HDR rate up to 60% [81]. Nevertheless, when HDR fails to repair the DSB, NHEJ occurs, which may lead to indel mutations that can cause gene dysfunction, ultimately worsening the phenotype. Moreover, targeting one allele over the other is challenging and can potentially lead to induction of the DSB in the healthy allele of heterozygotes and thereby exaggerate the disease symptoms.

All these attempts to overcome the challenges that go in hand with the use of genome editing push these technologies towards home stretch: clinical application.

## 5. Ethical Concerns

The rapid progress in genome editing technologies and their enormous popularity makes it even more important to discuss the ethical implication of genome editing in clinical therapy. Soon after the scope of opportunities emerging from the new genome editing tool, CRISPR/Cas9 became evident, and especially after the publications about attempts to edit human zygotes, concerns and the need for regulation were raised [58–60]. The National Academies of Sciences, Engineering and Medicine in 2015, prepared a statement on the use of human genome editing. The committee concluded that genome editing in basic science and future clinical use of the technologies in somatic cells are covered by the existing regulatory measures of gene therapy. The debate about germ-line modification on the other hand needs to be further addressed including the international scientific community as well as different perspectives from society. In their updated version from 2017, the committee concluded though that research on germ-line editing should continue, but clinical trials need to be evaluated with a strict risk and benefit consideration, being limited to untreatable and severe inherited diseases. Before moving on to alter the human genome permanently, consensus standards need to be developed and implemented. Fully understanding the risks of germ-line editing can lead the way to ensure a safe use of genome editing and enable an open productive discussion among science and society, especially in the most controversial field of genome editing, human enhancement.

## 6. Conclusion and Future Perspective

Genome editing tools, foremost CRISPR/Cas9, are one of the most promising technologies of our time. The progress made in basic research, the already implemented clinical therapies, and the potential of treating diseases based on genomic mutation are stunning. Genome editing has still a long way to go to be a safe and reliable therapeutic tool, but these technologies can open up a new era of medical treatment. Both, researchers and clinicians, need to utilize genome editing in a responsible manner in order to keep up a productive public discussion and thereby enable future patient-specific treatments (precision medicine).
